# 

**Published:** 1910-10-22

**Authors:** 


### CONTENTS.

| | PAGE |
|---|---|
| **The Meaning of Diathesis** | 93 |
| **Mob Psychology** | 94 |
| **Annotations—** Parliamentary—The War Office and the St. John Ambulance Association—Subdivision of Medical Labour | 95 |
| **Medical Opinion and Movement** | 96 |
| **Hospital Clinics—** Some Notes on the Treatment of Puerperal Septic Disease. By Knyvett Gordon, M.B. (Cantab.) | 99 |
| **Special Article—** On a Fatal Variety of Ulcerative Sore-Throat | 101 |
| **Medicine—** Ionic Medication | 103 |
| **Orthopædics—** Some Common Causes of Pain in the Foot—II. | 105 |
| **Pathology—** Tubercle Bacilli in the Fæces in Tuberculosis | 106 |
| **The General Practitioner's Column—** A Case of Hysterical Photophobia in a Child | 107 |
| The Treatment of Everyday Cases—Headache | 107 |
| **Practical Notes on Diagnosis and Treatment** | 108 |
| **News and Events of the Week** | 109 |
| **Appointments and Resignations** | 110 |
| **Obituary** | 110 |
| **The Week's Work—** The Fire at the Leeds Infirmary—Sickness Insurance—The Different Aspects—The Compulsory Insurance Scheme—"Workers' Hospital Funds"—This Week's Drug Market | 111 |
| **Parliament and Publications—** Hospitals in Ceylon—Hospitals in the Straits Settlements | 112 |
| **Special Institutional Article—** The Abuse of the Hospital and its Cure | 113 |
| **The Hospital World** | 118 |
| **Institutional Notes and News** | 119 |
| **Gifts and Bequests** | 120 |
| **Jottings** | 120 |

